# Effects of the Curing Regime, Acid Exposure, Alkaline Activator Dosage, and Precursor Content on the Strength Development of Mortar with Alkali-Activated Slag and Fly Ash Binder: A Critical Review

**DOI:** 10.3390/polym15051248

**Published:** 2023-02-28

**Authors:** Osama A. Mohamed

**Affiliations:** College of Engineering, Abu Dhabi University, Abu Dhabi P.O. Box 59911, United Arab Emirates; osama.mohamed@adu.ac.ae

**Keywords:** slag, fly ash, geopolymer mortar, sustainable construction, alkaline activator, curing environment, acid exposure, compressive strength

## Abstract

Reductions of green gas emissions and the reuse/recycling of industrial byproducts are important for the mitigation of the environmental impact of the construction industry. The replacement of ordinary Portland cement (OPC) is a concrete binder with industrial byproducts that possess sufficient cementitious and pozzolanic properties, such as ground granulated blast furnace slag (GBS) and fly ash. This critical review analyzes the effect of some of the most critical parameters on the development of the compressive strength of concrete or mortar that consists of combinations of alkali-activated GBS and fly ash as binders. The review includes the effects of the curing environment, the proportions of GBS and fly ash in the binder, and the concentration of the alkaline activator on strength development. The article also reviews the effect of exposure as well as the age of samples at the time of exposure to acidic media on the development of concrete strength. The effect of acidic media on mechanical properties was found to depend not only on the type of acid but also on the alkaline activator solution, proportions of GBS and fly ash in the binder, and the age of the sample at the time of exposure, among other factors. As a focused review, the article pinpoints important findings such as the change in compressive strength over time when mortar/concrete is cured in an environment that permits the loss of moisture versus curing in a system that retains the alkaline solution and keeps reactants available for hydration and the development of geopolymerization products. The relative contents of slag and fly ash in blended activators have a significant impact on strength development. Research methods used include a critical review of the literature, a comparison of reported research findings, and identifying reasons for agreement or disagreement of findings.

## 1. Introduction

Alkali-activated concrete is developed when selected source materials, also known as precursors, are activated utilizing an alkaline material, such as NaOH or Na_2_SiO_3_, to act as a binder for concrete/mortar [1]. Source material/precursors that were used as activated binders include those of geological origins such as kaolites or clay, or an industrial by-product, such as fly ash, ground granulated blast furnace slag (GGBS), or silica fume [2]. Mine tailings (MTs) were also found to represent promising precursors for alkali-activated material, not only to save landfill by reusing the byproducts of the mining industry, but also due to the favorable properties of the produced concrete and mortar [3]. Similarly, agro-industrial waste, such as that from sugar cane and rice, may be recycled and reused for the production of alternative cementitious materials to produce concrete and mortar [4]. The source material, or precursor, typically contains a substantial amount of silicon and aluminum as fundamental components of their chemical composition that will eventually form the binding gel [5]. Because the chemical reaction that takes place from the activation of the precursor to the formation of the binding gel is a polymerization or geopolymerization process, the material is often called a geopolymer [6]. Geopolymer concrete binders may be a combination of two or more geological sources or industrial by-products, such as GGBS and fly ash, or other combinations [7]. The fly ash precursor emphasized in this article is ASTM class F [8], which is derived from bituminous coal, but limited discussion on ASTM class C (high-calcium fly ash) is also provided [9]. Similarly, when referenced in this article, GGBS refers to slag that is compliant with ASTM C989/989M [10]. It is important to note that fly ash that is not ASTM compliant may produce a geopolymer with variable mechanical properties [11], to which the findings of the present review do not apply. Alkaline liquids that are commonly used to activate the binder are developed from soluble alkaline metals such as sodium or potassium or may come from combinations of these metals such as sodium hydroxide and sodium silicates [12]. One advantage of combining GGBS with fly ash as a binder is that GGBS offers early strength development while fly ash is typically responsible for the longer-term strength of concrete [13]. The activation of fly ash to be used as sole concrete/mortar requires heating to set and develop strength in a reasonable time, a shortcoming that impedes its use for practical applications. Therefore, blending fly ash with the fast-reacting GGBS offsets this problem and contributes to fly ash activation. Mortar developed utilizing alkali-activated fly ash (AAF) as a sole binder exhibited lower chloride binding capacity and diffusion resistance than ordinary Portland cement (OPC) [14,15], a shortcoming that may be mitigated by blending fly ash with slag. This article reviews the current state of knowledge of geopolymer concrete developed using combinations of fly ash and slag, being amongst the most commonly produced industrial by-products that would otherwise need to be landfilled, if not reused. Several published reviews cover numerous topics on alkali-activated material including the performance of various precursors, short-term properties, durability, and strength. The broad coverage may lack depth on essential individual topics. This review article, however, focuses on critical factors influencing the strength development of mortar and concrete with alkali-activated binders. This article was developed based on a critical analysis of published literature to identify common findings related to the most important factors affecting the strength development of concrete and mortar with alkali-activated slag and fly ash binders. The databases used included but were not limited to ScienceDirect, American Society of Civil Engineers (ASCE), and ResearchGate. Some of the keywords used in the database searches included the following: alkali-activated binder, geopolymer, fly ash, ground granulated blast furnace slag, compressive strength, water curing, air curing, and acid exposure. Explanations of findings that are common or different in published literature were presented and analyzed. 

Future work should be dedicated to investigating other essential aspects of alkali-activated mortar and concrete, such as shrinkage, chloride penetration resistance, and resistance to degradation due to sulfate attack or exposure to acidic media. Future research opportunities also include the reuse of other precursors and activators that are environmentally friendlier and/or offer further opportunities for the reuse and recycling of industrial and agricultural byproducts. 

## 2. Geopolymerization Process, Reaction Products, Microstructure, and Porosity

Geopolymers are generally non-crystalline (amorphous) aluminosilicate materials that form a cross-linked covalently bonded network of alumina and silica tetrahedral [16]. Numerous alkaline activators were used successfully in the hydrous or anhydrous form to activate various precursors, such as sodium silicate (Na_2_SiO_3_), sodium hydroxide (NaOH), or a combination of these materials. Although this article focuses on alkaline activators, acid-based activators such as phosphoric acid are promising alternatives in terms of producing geopolymer mortar and concrete of superior properties. An elaborate discussion of geopolymer mortar and concrete with acidic activators can be found in the literature [17].

Alkaline activators, the activation method reviewed in this article, are composed of cations from the first two groups in the periodic table, such as Ca^2+^, Na^+^, and K^+^, along with alkali components such as ${\mathrm{SiO}}_{3}^{2-}$, ${\mathrm{CO}}_{3}^{2-}$, and OH^−^ [18]. NaOH and Na_2_SiO_3_ are the most popular alkaline activators as they are effective in producing binding gel, are relatively inexpensive, and are easy to prepare. Na_2_SiO_3_, however, is more expensive than NaOH, partly due to the high energy required in the preparation process [18]. Na_2_SiO_3_, on the other hand, contributes to the formation of binding gel in two ways, firstly through the provision of the alkaline environment for the dissolution of the precursor, and secondly through the supply of Si that is an integral part of the binding gel [13].

The reaction rate and development of the microstructure of slag activated using NaOH is faster and increases with the activator concentration during the first 24 h compared with when it is activated using Na_2_SiO_3_ [19]. Na_2_SiO_3_, on the other hand, has a longer induction period and reacts at a slower rate with slag at an early age up to 24 h but reaches a significantly higher compressive strength by the age of 28 days compared to activation using NaOH [19]. The induction phase, which is the second of the slag hydration phases, is much shorter, possibly a few minutes, when the activator is NaOH [20]. The binding gel C-S-H begins to develop during the induction phase of the slag hydration and is fundamental to the development of early strength in alkali-activated mortar.

Water plays a very important role in the polymerization of alkali-activated composites. It simultaneously acts as a medium of dissolution and for the transport of silicate and aluminate ions. Water is needed in the dissolution process to foster the polymerization process and form monomers such as SiO(OH)_2_, Si(OH)_4_, and Al(OH)_4_, or oligomeric species [21]. It is also needed to initiate the polycondensation process. Some of the mixing water, whether it is part of the alkaline activator solution or added separately, is lost through evaporation, a process that continues after the hardening of the polymeric matrix. However, some of that water does not evaporate and remains trapped within the hardened polymeric gel system. In fact, the trapped non-evaporable water remains in the gel network even after the application of a temperature higher than 1000 °C. The non-evaporable water also supports strength development at an advanced mortar age as it continues to dissolve unreacted Al^3+^ and Si^4+^ compounds [22].

In OPC-based concrete and mortar, calcium silicate hydrate and calcium hydroxide are the main reaction products. However, different types of gel develop when GGBS/fly ash is activated using alkaline activators, such as C-A-S-H, C-N-A-S-H, and N-A-S-H [23]. The development of these polymerization products in terms of presence, quantities, and rate of the process depends on the type(s) of precursor(s), curing conditions/temperature, and type of activator, along with other factors. For instance, lower Na^+^ levels were found in unsealed-cured alkali-activated mortar samples compared to samples that were sealed during curing, as will be further detailed in this article. Evaluation of samples cured for 1 day using X-ray diffractograms reported the absence of N-A-S-H gel from hydration products of GGBS/fly ash geopolymer mortars. However, C-A-S-H was detected clearly in AAFS binder pastes after 1 day of curing [24]. The GGBS:fly ash binder ratio was 80:20, which resulted in the supply of a large amount of Ca^2+^ that combined with Al and Si ions to develop C-A-S-H gel at each of the two curing temperatures (20 °C and 40 °C) examined in the study. The activator used was a mixture of NaOH and Na_2_SiO_3_ solutions. In fact, it was reported that the presence of Ca^2+^, typically supplied by slag, hinders the formation of less compact sodium-based zeolites [25] and mitigates their impact on the compressive strength. The absence of zeolites, which are intrinsically microporous, from the reaction products of AAS was also confirmed by Oh et al. [26]. The main hydration products of AAS were crystalline phases consisting of C-S-H(I) and hydrotalcite. 

Microstructure analysis conducted by Goa [27] indicated that when up to 2% microsilica was added to a blended precursor consisting of slag and fly ash binders, the fundamental polymerization product was C-A-S-H, irrespective of the relative content of slag to fly ash included in their study. 

The hydration of GGBS provides calcium to the alkali-activated system, in addition to hydrated calcium silicate hydrate (C-S-H) gel. The alkali-activation of fly ash on the other hand produces the aluminosilicate gel N-A-S-H [28]. The two gels produce a significantly more compact microstructure than OPC-based gel [13]. In addition, the co-existence and interaction of these two gels produce reaction products with a greater extent of cross-linking. 

When the alkaline activator of GGBS is silicate-based (such as sodium silicate), C-A-S-H gel represents the primary polymerization product. On the other hand, silicate activation of fly ash is dominated by the formation of N-A-S-H gel, which has a more porous structure, leading to easier transport through mortar/concrete according to Ismail et al. [28,29]. The hardened matrix formed by C-A-S-H gels is typically denser and less porous than that formed by N-A-S-H [30]; therefore, increasing the content of fly ash leads to an increase in the volume of permeable voids, especially when fly ash exceeds 50% of a binder composed of a combination of slag and fly ash [28]. 

In mortar with an alkali-activated slag and fly ash (AASF) binder with a slag content greater than 50%, Ismail et al. [29] identified N-C-A-S-H as a fundamental binding material, where Na and Al were incorporated into calcium–silicate–hydrate. However, in AASF binder, where the GGBS content ranged from 10% to 30%, Lee et al. [31] reported the hybrid phase C-N-A-S-H with a high Ca content as the main product in mortar with the AASF binder system rather than the discrete phases of N-A-S-H and C-A-S-H. 

Jang et al. [32] reported that in mortars with AASF binders, a higher content of slag produced a denser matrix of hydration products and polymerization material. Furthermore, the higher the content of slag in GGBS/fly ash blends, the higher the compressive strength. However, increasing the slag content accelerates the chemical reaction [33] and causes rapid setting and cracking due to autogenous shrinkage, especially in mortar/concrete with 70% and 100% slag contents, which agrees with the findings of Li et al. [34].

The increased porosity of mortar/concrete is detrimental to durability, while tortuosity has the opposite effect. Tortuosity is an indicator of the length of the transport path through concrete/mortar. Increasing the slag content decreases segmented porosity and increases diffusion tortuosity. In addition, porosity decreases and tortuosity increases with the sample curing age [35]. The increase in tortuosity with curing age is particularly evident in binders with a high slag content (≥50%) where more of the dense and compact C-A-S-H is contributed, leading to highly reduced porosity of the total binding gel. A decrease in the fly ash content in AASF also means that a correspondingly smaller amount of the porous N-A-S-H gel contributes to the total binding phase. The strength of mortar with an AASF binder and a slag content of 25% or less is dominated by N-A-S-H gel, which contributed to the binder gel by the activation and geopolymerization of fly ash [35]. When the fly ash content was 90% fly ash (slag = 10%), Pan [36] described the reaction product as N-(C)-A-S-H, which is a binding phase characterized by the dominance of fly ash through Na and the presence of calcium from slag. N-A-S-H and N-(C)-A-S-H have similar structural properties and thermal stability up to a temperature of 300 °C. However, at an elevated temperature of 600 °C, the two products are different as N-A-S-H develops higher strength due to the heat-stimulation of the fly ash reaction, while N-(C)-A-S-H decreases in strength [36]. N-A-S-H gel does not have the same strength and density of the pore system that characterizes the slag-based C-A-S-H gel.

In mortar with an AASF binder, higher contents of slag result in higher tortuosity and lower porosity compared to binders that are richer in fly ash [35]. Increased tortuosity, which prolongs the travel path of deleterious materials to reinforcing steel, enhances the durability of mortar/concrete with an AASF binder. The enhanced durability afforded by lower porosity is particularly important in alkali-activated geopolymers as they lack portlandite, the pH buffering phase that usually provides corrosion protection when the binder is OPC. The proportions of slag and fly ash in the total binder are important in determining the actual performance in terms of durability and strength due to the difference in the properties of C-(A)-S-H and N-A-S-H gels. For instance, C-A-S-H and N-A-S-H may differ in their capacity to bind alkalis, carbonations, and chlorides [35]. Table 1 shows a summary of the main geopolymerization products of alkali-activated slag and fly ash binders, along with some of their essential properties. 

In mortar with AAF, the porosity of the gel system depends on the activator type. The average pore diameter was the lowest in fly ash activated using sodium hydroxide and the highest in mortar with fly ash activated using a combination of NaOH and KOH alkaline solutions [37]. The average pore diameter was found to correlate with the compressive strength development, as well as the stability of mass after submersion in sulfuric or acetic acid. Mortars with smaller median pore diameters demonstrated higher stability and lower mass loss after fly ash-based samples were subjected to sulfuric acid [37]. In mixes with a w/b ratio of 0.3 and Na concentration of 8%, fly ash-based mortar developed a higher average pore size diameter when potassium hydroxide was added to sodium hydroxide compared to fly ash activation using sodium hydroxide alone [37]. The sodium silicate (Na_2_SiO_3_) activator produces mortar with a median pore size relatively larger than that of sodium hydroxide (NaOH) but much smaller than that of the combined NaOH and Na_2_SiO_3_ activator solutions. Therefore, it is likely that fly ash-activated utilizing sodium silicate would produce mortar that is more durable with enhanced resistance to sulfuric acid attack compared with potassium hydroxide. 

**Figure 1:**
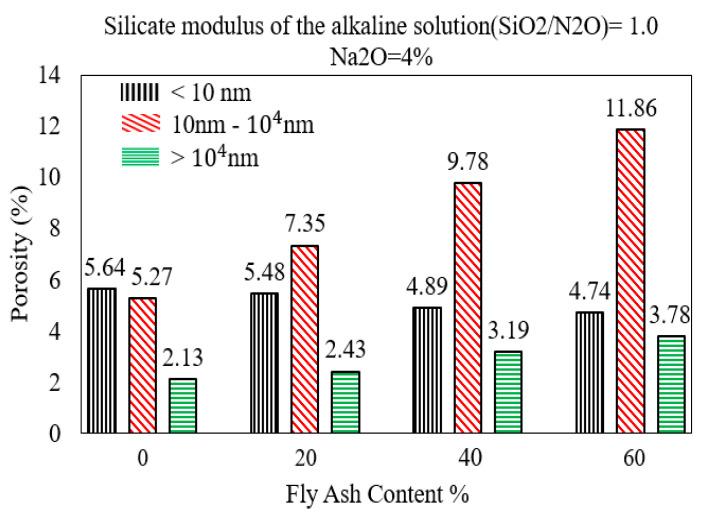
Effect of the fly ash content on the porosity of AASF mortars at the age of 28 days [38].

Several factors generally affect the porosity of mortar with an AASF binder including the silicate modulus of the alkaline activator (SiO_2_/Na_2_O), the dosage of the alkaline activator (Na_2_O), and the relative content of fly ash. The total porosity of mortar with an AASF binder decreases significantly with an increase in the silicate modulus and an increase in the dosage of the activator. In addition, the total porosity increases when the fly ash content is increased [38]. When porosity changes for any reason, such as an increase in the content of fly ash, it is manifested as a variation in the volume of pores in the range of 10 to 10^4^ nm as shown in Figure 1, while the volume change of pores outside of this range is generally limited [38]. A pore system within mortar/concrete sample is determined by the reaction products that form the binder gel. The sodium-based N-A-S-H gel, which is a less compact product, is attributed to the geopolymerization of fly ash and produces higher porosity. The calcium-based C-A-S-H gel, which is dense and compact, is attributed to the geopolymerization and hydration of GGBS that produce a system with lower porosity [38,39].

**Figure 2:**
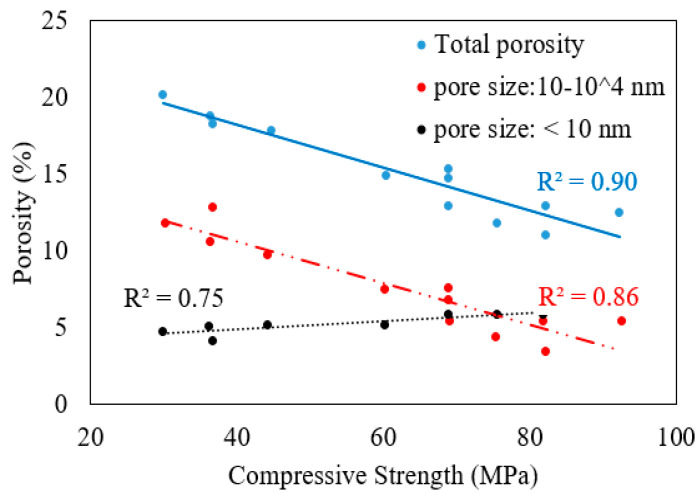
Effect of pore sizes on the strength development of AASF mortar [38].

The compressive strength of mortar with an AASF binder is strongly correlated with the total porosity [38] and the volume of pores whose sizes range from 10 to 10^4^ nm. Compressive strength decreases with an increase in overall porosity and an increase in the percentage of pores whose sizes fall in the range of 10 to 10^4^ nm as shown in Figure 2 [38]. In addition, a trend exists where compressive strength increases with the percentage of air voids that are smaller than 10 nm and decreases with an increase in the percentage volume of larger air voids (>10^4^ nm).

## 3. Effect of Acid Exposure on the Integrity of the Mass and Mechanical Properties

In conventional concrete with an OPC binder, exposure to many types of acids, especially for extended periods, may cause a substantial reduction in mass and strength. Concrete is naturally alkaline, which keeps the binding gel stable. When concrete remains in contact with a highly acidic solution, the alkalinity of concrete/mortar decreases, possibly below the stability limit of the binding gel. The acidity of a solution is typically quantified by the magnitude of its pH, a measured quantity that is an indicator of the amount of dissociated hydrogen protons (H^+^). Strong acids, such as sulfuric and hydrochloric acids, become completely dissociated into their ionized form in aqueous solutions. Weak acids, such as organic acids, dissociate only partially into their ions in aqueous solutions [40]. If the pH of concrete is lower than the stability threshold of the binding silicate–hydrate gel, calcium is removed from the structure and the hydrogel becomes highly porous. The calcium ejected from the gel structure develops calcium salts of the particular acid, in addition to other hydrogels. After exposure to strong acids, concrete/mortar with an OPC binder experiences damage in the form of scaling and softening caused by the decomposition of calcium hydroxide and the development of gypsum. 

Organic acids, including but not limited to acetic acid, cause a higher degradation in mortar/concrete compared to “strong” acids because of the high solubility of the hydration and polymerization reaction products and because of the acid buffer action [40]. However, when alkali-activated material is subjected to saturated strong acid solutions, such as sulfuric (H_2_SO_4_) acid, the resulting calcium-based salts, such as gypsum, do not accumulate on the surface of the sample due to their lower solubility [40]. 

Larreur-Cayol et al. [41] reported that the solubility of calcium salts in organic acids was a fundamental contributor to the severity of damage induced in the mortar matrix with an alkali-activated binder. Amongst the three organic acids tested, citric acid demonstrated the highest kinetics and induced the most severe deterioration in the paste compared to intermediate damage due to acetic acid and moderate damage due to tartaric acid. Therefore, prolonged exposure of OPC-based concrete to a deleterious environment that is rich in acids, such as a sewerage system, may cause an extensive reduction in strength [42,43]. In the case of exposure to sulfuric acid, degradation in the mass of mortar increases with the total OPC binder content [44]. 

When the sole binder is AAS, exposure to sulfuric acid has a limited effect on the mass and strength of mortar. The formation of gypsum, the reaction product, is limited to the exterior of the samples up to a limited depth. This is because of the dense nature of the slag-based matrix that impedes acid transport and prevents propagation to the inner core [45]. Similarly, when AASF is the binder of mortar/concrete, increasing the content of slag decreases the deleterious effect of the exposure to sulfuric acid [1]. Degirmenci et al. [46] reported that mortar with an AAS binder demonstrated the best resistance to degradation after sulfate and sulfuric acid attacks in contrast with mortar where the binder contained various amounts of fly ash. This may be due to the refined pore system formed by the hydration products of GGBS, which obstructs the progression of sulfuric acid. When mortar with an AAFS binder is submerged in sulfuric acid, a decrease in the Al/Si ratio was observed in the outer segments of the samples that were in direct contact with the acid compared to the inner core. Aiken et al. [47] demonstrated that increasing the content of slag in mortar with an AASF binder decreased the porosity of the matrix. Despite the lower porosity, it was noted that the reaction products were still degraded by sulfuric acid attack. The resistance of mortar/concrete to degradation induced by acid exposure depends on the acid type, the relative proportions of fly ash and slag in the binder, and the alkaline activator type and concentration. 

When mortar with fly ash binder was exposed to sulfuric acid with a concentration as low as 3%, porosity was severely compromised due to the leaching of gypsum that caused an increase in the pore system [48]. Furthermore, when subjected to MgSO_4_ solution for 10 years, fly ash-based mortars developed fewer cracks and limited swelling compared to mortar with an OPC binder [48]. The reaction of sulfate ions emanating from MgSO_4_ with calcium aluminate hydrate and portlandite (Ca(OH)_2_) led to the development of gypsum and ettringite, leading to swelling and subsequent cracking in OPC-based mortar. However, the penetration of sulfates into fly ash-based mortar was deeper than that into OPC-based mortar. This is likely due to the noncompact pore system that characterizes N-A-S-H, the dominant geopolymerization product of AAF binders [2]. Nonetheless, Thokchom et al. [49] reported that mortar with AAF as a sole binder outperformed OPC and experienced very limited weight loss after exposure to 10% sulfuric acid for 18 weeks. The geopolymer samples lost alkalinity completely after 18 weeks of immersion in sulfuric acid, along with a maximum of 52% loss in compressive strength. It is possible that heat curing, which is necessary for AAF-based mortar, accelerated the development of the reaction products, and the small amount of calcium in fly ash played a role in the relatively enhanced degradation resistance. 

**Figure 3:**
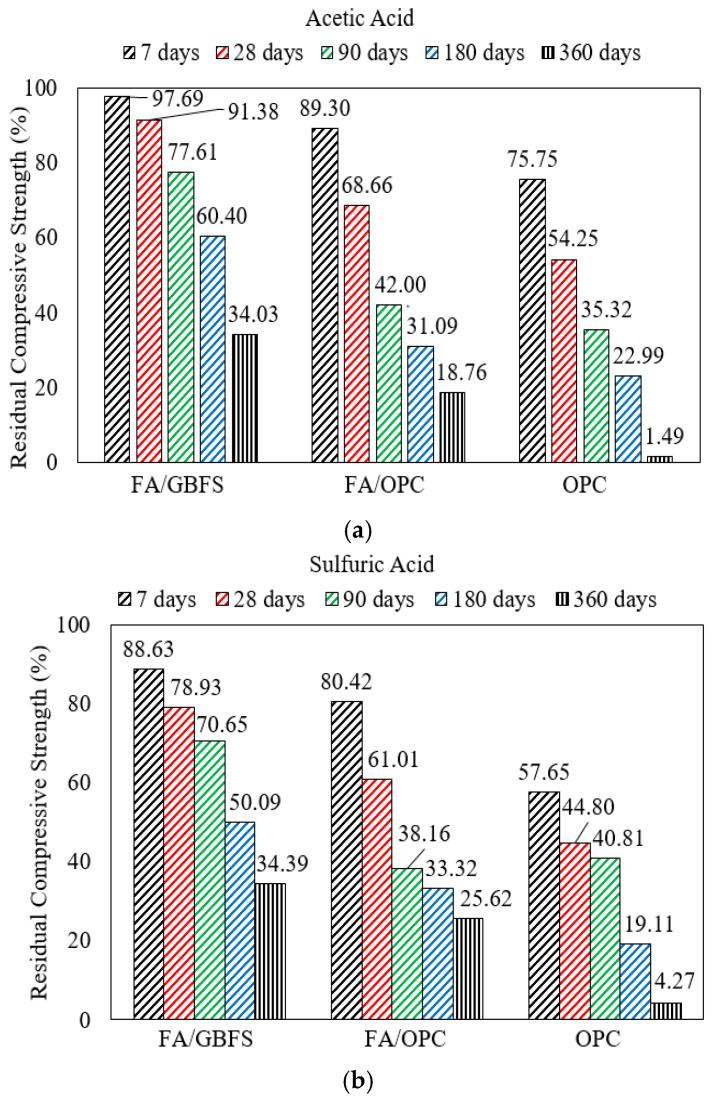
Strength degradation of mortar with various binders including AASF with fly ash:slag = 4:1, fly ash:OPC = 4:1, or 100% OPC in (**a**) acetic acid (**b**) sulfuric acid [50].

In general, replacing even a smaller amount of fly ash with GGBS improves the acid resistance of alkali-activated mortar. While mortar with an AAF binder remains vulnerable to acid attack, it still outperforms OPC-based mortar. In a study by Valencia-Saavedra [50], mortar with a high fly ash content (fly ash:GGBS ratio of 80:20) lost 66% of its compressive strength after 360 days of immersion in 1 M acetic acid (CH_3_COOH), while OPC-based mortar lost nearly 98% of its compressive strength as shown in Figure 3a. Similar improved resistance to sulfuric acid was exhibited by mortar with an AASF binder compared to OPC-based mortar after 360 days of exposure as shown in Figure 3b. Therefore, extended exposure (360 days) to highly acidic CH3-COOH or H_2_SO_4_ resulted in the complete destruction of OPC-based mortar, while mortar containing a high content of fly ash was able to retain more strength after one year of exposure [50].

Some studies reported an increase in the weight of mortar with an AASF binder after a certain number of days in acid. One study reported that alkali-activated specimens with a fly ash/GGBS ratio of 80/20 that were exposed to H_2_SO_4_ experienced a relatively small increase in weight after 90 days of exposure [50]. The small increase in weight may be due to the low solubility of calcium sulfates that deposit on the pore system of the material and densify the structure [51]. A similar increase in weight after the exposure of mortar to sulfuric acid was reported by other investigators as well [52,53,54,55]. In the study by Valencia-Saavedra et al. [50], the highest reduction in the mass of mortar in organic or strong acids at all ages was exhibited by OPC-based mortar, while geopolymer samples exhibited better resistance. This is due to the higher content of calcium in OPC-based mortar compared to AASF binders.

**Figure 4:**
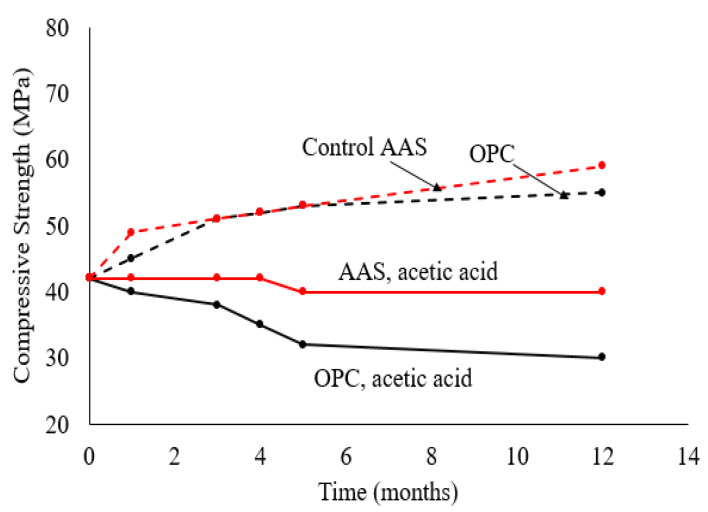
Compressive strength development of mortar with AAS or OPC binders in acetic acid compared to immersion in water [56].

Mortar prepared using 100%GGBS as a binder outperformed OPC-based mortar/concrete when submerged in sulfuric acid, largely due to the relatively lower calcium content in GGBS compared to OPC [2,5]. After immersion in acetic solution (pH = 4) for one year, mortar with an AAS binder lost 33% of its compressive strength while OPC-based mortar lost 47% of its compressive strength [56]. This was attributed to decalcification and formation of highly soluble calcium salts, such as calcium acetate, which may leach and leave a matrix with lower density [56]. As the calcium content is higher in OPC, the mortar contains a larger amount of decalcified C-S-H that is soft and does not contribute to the compressive strength. The calcium acetate formed is soft, which may contribute to increased weight if it does not leach out, but does not contribute to the compressive strength. It is worthy to note, however, that the data do not indicate a decrease in the strength of samples immersed in acetic acid solution over time up to four months of immersion. However, the strength of samples immersed in acidic solution was much lower compared to baseline samples that were immersed in water as shown in Figure 4.

Calcium-based products, such as gypsum, were identified as an outcome of mortar/concrete deterioration due to acid attacks. Calcium is abundant in OPC paste in the form of C_2_S and C_3_S, portlandite (Ca(OH)_2_]), and C-S-H. When mortar/concrete is subjected to acetic acid, for instance, the calcium-containing compounds react with the acetic acid, producing calcium acetate. Similar to many compounds that result from an attack by organic acid, calcium acetate is soluble and may leach out of the paste leaving decalcified C-S-H, which is soft and has negligible structural properties. The improved performance of concrete with an AAS binder when subjected to the acid solution was ascribed to the relatively lower Ca in slag compared to OPC paste. After exposure to acid, alkali-activated slag paste becomes rich in Si and depleted in Ca. When exposed to acetic acid, a degradation in compressive strength occurs and may be attributed to the replacement of the stiff C-S-H with decalcified C-S-H that does not have structural stiffness, as well as soluble calcium acetate.

In general, mortar and concrete with AAF binders performed better when exposed to strong or organic acid compared to those based on OPC. Nonetheless, significant degradation and a decrease in strength were observed when fly ash was activated using a mixture of NaOH and KOH alkaline activator solutions. The deterioration was attributed to the depolymerization of the aluminosilicate gel and the development of zeolites as a result of acid exposure. Zeolites are typically porous aluminosilicates with a crystalline structure and micropores in the range of 0.2 to 2 nm that are interconnected through channels. Fly ash-based polymerization products that were produced when the alkaline activator was sodium hydroxide exhibited a more stable cross-linking and therefore were able to provide better resistance to deterioration due to sulfuric acid attack in contrast with sodium silicates or combinations of sodium silicates and sodium hydroxide [37].

When exposed to 5% sulfuric acid, fly ash-based geopolymer activated using sodium hydroxide with 8% Na concentration exhibited the best performance with 17.5% strength degradation after 4 months of immersion in the acid, followed by the stability of the specimen and the maintenance of strength. Similar samples activated using NaOH and KOH blended solutions lost nearly 89% of their strength after 60 days, while samples activated using sodium silicate lost 82% after the same number of days. On the other hand, identical OPC-based mortar samples deteriorated entirely after the first month of acid immersion, while mortar with OPC + FA binders experienced a 77% decrease in strength and was highly deteriorated [37].

Resistance to the acidic media of fly ash-based geopolymers depends on the alkaline activator type and on the acidic solution itself. In the same study [37], fly ash-based geopolymer samples subjected to 5% acetic acid performed better when the activator was a combination of NaOH and KOH solutions compared to acid resistance when the activator used was only NaOH. However, compressive strength loss after 6 months in samples activated using sodium and potassium hydroxide was about 38.3%, while samples activated using sodium hydroxide lost about 45% of their strength during the same period.

As indicated earlier, the superior performance of mortar with AAF or AAS binders in acidic media, in contrast with OPC-based mortar, was attributed in some studies to the higher content of calcium in OPC. Allahverdi and Skvara [51] attributed damage to mortar containing equal proportions of fly ash and slag binders and immersed in sulfuric acid to the ejection of aluminum when acid protons attacked the polymeric Si-O-Al bonds in the aluminosilicate framework. The process of aluminosilicate ejection leads to shrinkage cracks through which sulfate anions diffuse to react with calcium to form and deposit gypsum [51]. In high-concentration sulfuric acid solution (pH ≈ 1.0), the formation and deposition of gypsum inside the corroded matrix provide a protective layer, which inhibits further deterioration of the matrix [51].

After immersion in an aggressive environment (sea water or sodium sulfate solution) for up to 365 days, fly ash-based geopolymer developed relatively higher strength when activated utilizing a combination of NaOH and Na_2_SiO_3_ solutions compared to samples activated using sodium hydroxide alone [57]. In the study by Fernandez-Jimenez [57], mortar samples with AAF binders were heat-cured at 85 °C for 20 h and 99% RH before exposure to an aggressive environment. 

When immersed in HCl solution (pH = 1.0) for up to 90 days, mortar with an AAF binder activated using a combination of NaOH (85%) and Na_2_SiO_3_ (15%) solutions or NaOH alone lost 23% to 25% in compressive strength. However, the use of NaOH and Na_2_SiO_3_ mixtures to activate fly ash-based mortar produced mortars with higher strength at each curing age from 7 days to 90 days compared to the use of NaOH as the sole activator. After 90 days in HCl solution, OPC-based mortar lost 47% compressive strength, performing poorer than fly ash-based samples for either activator type [57]. After immersion in solution for 90 days, fly ash-based mortar lost up to 4.2% of its mass, while OPC-based samples lost 9.8% of their initial weight.

In mortar with an AAS binder, the degradation of mass and compressive strength was associated with the dealumination of the aluminosilicate structure. Exposure to 5% acetic and sulfuric acid solutions creates fissures in the gel by breaking the bonds, such as Si-O-Al, which ultimately leads to the loss of mass [37].

The deterioration of mortar due to organic acids, such as those found in agri-food effluents, depends not only on the contents of slag and fly ash in a blended binder but also on the type of organic acid. Aiken et al. [58] reported that a change in the concentration of acetic and lactic solutions from 0.1 mol/L to 0.52 mol/L had little impact on compressive strength. Mixes with a higher fly ash content had relatively better resistance to strength degradation in acetic solution, while mixes with a high slag content had higher resistance to degradation in lactic acid. The overall reduction in the compressive strength of mortar with slag–fly ash binders ranged from 31% to 49%, depending on the type of organic acid and the proportions of slag and fly ash [58]. Mortar immersed in acetic acid (pH 3.2) for 100 days performed better in terms of compressive strength retention and the stability of mass when the binder was an AAS binder compared with AAF, while both precursors (slag and fly ash) outperformed OPC [59]. The decalcification of the geopolymerization products was identified as the dominant degradation mechanism under acid attack. Nonetheless, the relatively superior performance of mortar with AAS compared to AAF under attack by acetic acid is attributed to the denser microstructure and lower permeable porosity of slag.

The reason for the relatively higher vulnerability of mortar with a high slag content in acetic solution is the higher solubility of calcium acetate. However, as the slag content increased from 20% to 70%, the reduction in mortar compressive strength in acetic acid increased from 38% to 43%. This is because the higher content of calcium in slag leads to the development of additional quantities of the highly soluble calcium acetate [6]. AASF mortar with a slag:fly ash ratio of 7:3 exhibited the best resistance to deterioration when exposed to lactic acid for 8 weeks [58]. A similar ratio of slag/fly ash of 75/25 was reported to exhibit higher strength development compared to other ratios and was attributed to effective pore filling, cross-liking, and the possible activation of fly ash by the slag itself [1,2]. In Aiken’s study [58], sample curing was continued for 21 days after demolding, followed by the immersion of mortar cubes under water for an additional 28 days. Samples were then immersed in an organic acid solution. It was shown that the curing regime after demolding significantly affected the resistance of alkali-activated mortar to strength degradation in acidic media [1,2].

The sensitivity of mortar with an AAS binder to degradation due to acetic acid (CH3COOH) was also reported by Bernal et al. [60]. Alkali-activated slag mortar retained much higher strength compared to OPC-based mortar after 150 days of exposure to acetic acid (pH = 4.5). Although CH3COOH is not a strong acid, i.e., it dissociates slowly compared to stronger acids, such as sulfuric acid, it resulted in the degradation of the mortar samples. This was attributed to the higher solubility of calcium acetate produced during the acid attack on the matrix. The high calcium content in OPC and slag reacted with acetic acid and produced higher amounts of soluble calcium acetate in OPC-based mortar and smaller amounts in slag-based mortar. The alkaline activator used in the study was sodium silicate, and samples were cured at RH greater than 90% for 60 days before immersion in an acidic medium.

A study by Bernal et al. [60] showed that mortar with a sodium silicate activator gained strength while exposed to HCl, HNO_3_, and H_2_SO_4_ for 150 days, which the investigators attributed to the maturity of samples in the inner core. When compared to mortar samples immersed in water, alkali-activated slag-based mortar samples gained less strength after 150 days. Alkali-activated slag-based mortar outperformed OPC in terms of strength development when cured in water or exposed to mineral acids. The findings of Bernal et al. [60] are similar to the data reported by Mohamed [1], where slag-based samples submerged in sulfuric acid (pH = 1.0) continued to gain compressive strength over time, but these samples were exposed to acidic medium 24 h after casting. This allowed the curing water and alkaline activator to remain available for the geopolymerization reaction and for strength development to continue over time. The high curing humidity (>90%) for 60 days before immersion in the Bernal study [60] may have provided a similar environment where the loss of water was minimized before immersion in an acidic medium.

Table 2 shows the change in mass and strength due to the exposure of geopolymers, alkali-activated mortars, or OPC-based mortar to acetic or sulfuric acid. It is clear that exposure to acidic media leads invariably to the degradation of mass and compressive strength. However, the extent of the degradation varies with the acid type, alkaline activator, curing conditions, and binder type. Table 2 shows that fly ash-based geopolymer subjected to acetic acid for two months experienced similar degradation when the activator was a NaOH (45%) or Na_2_SiO_3_ (−46%) solution. However, fly ash-based geopolymer activated using Na_2_SiO_3_ solution experienced an 82% decrease in compressive strength due to immersion in sulfuric acid, much higher than the 46% decrease experienced by the fly ash-based mortar samples when immersed in acetic acid for two months. However, when the activator used was NaOH, the effect on the same fly ash-based mortar samples was reversed and acetic acid caused higher degradation in strength (45%) than sulfuric acid (10.4%), after two months of exposure.

## 4. Effect of the Curing Environment/Method on the Durability and Strength of Geopolymer Concrete

**Figure 5:**
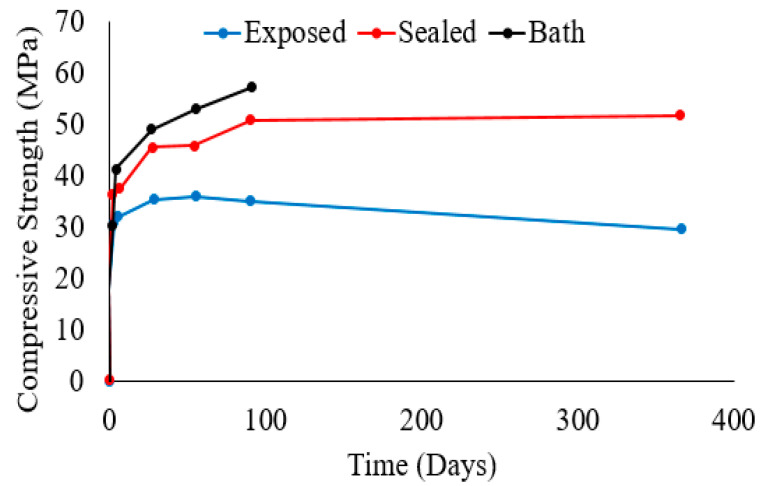
Strength evolution of concrete cylindrical samples with an AAS binder cured in air, sealed, or in a water bath [62].

After alkali-activated samples are cast, the retention of the mixing liquid containing the alkaline activator is essential for polymerization reactions and strength development. Concrete cylinders with slag binder activated using sodium silicate powder were tested by Collins and Sanjayan [61,62] under different curing conditions. Mortar exposed to air started to decrease in strength after the age of 28 days, while samples that were either sealed after casting or placed in a water bath until the test day continued to increase in strength until the age of 365 days, as shown in Figure 5. Samples cured by exposure to ambient conditions at 23 °C and 50% RH had 41.4% and 53.5% lower strength than cylinders that were sealed and bath-cured, respectively [62]. Mohamed et al. [2] also reported that mortar samples with AAS binders that were cured in the air exhibited a decrease in compressive strength after the age of 28 days and exhibited significantly lower strength by the age of 90 days. Collins and Sanjayan [62] noted the existence of shrinkage microcracking and higher total porosity in samples cured in air. After 365 days of curing, sealed samples has about 17.5% lower compressive strength compared to bath-cured samples, indicating the loss of mixing water and alkaline solution, although to a lesser extent than the exposed samples [62]. 

Mortar samples with a slag binder activated using powdered sodium silicate developed better slump characteristics, as opposed to the rapid setting that occurs when the sodium silicate activator is in liquid form. This is due to the slow release of alkalis into the system when the activator is in powdered form compared to liquid form [61]. In addition, when cured in a water bath, mortar with slag activated using an anhydrous sodium silicate activator developed a higher compressive strength than that of OPC-based mortar. It also outperformed mortar with a slag binder activated with sodium hydroxide and sodium carbonate in liquid form [61].

Whether mortar with an AASF binder is cured in air or water, strength development depends on additional factors, such as the relative contents of slag/fly ash, as well as solution alkalinity [1]. Data published by Wardhono et al. [63] indicate that samples cured in water with an AASF binder containing equal percentages of slag and fly ash exhibited a higher compressive strength after curing for 28 days, in contrast with mortar with an AASF binder, and slag contents of 100%, 90%, 80%, 70%, and 60%. In that study, the concentration of the NaOH solution was 15 mol/L, which makes the alkalinity of the solution relatively high. This is compatible with the findings of Mohamed et al. [2], where a similar mortar with an AASF binder exhibited the highest compressive strength after water curing for periods up to 90 days when the concentration of the NaOH activator solution was 16 M. In that study, NaOH molarity ranged from 10 M to 16 M. When NaOH molarity was 15 M, mortar with a slag content ≥70% developed a lower level of strength between the ages of 7 days to 90 days [63].

A high amount of calcium in the precursor, as in the case of slag, leads to early strength development, often without heat curing, due to the rapid release of calcium into the solution. This may also lead to flash setting in mortar with high-calcium fly ash, such as ASTM class C [64]. Therefore, in mortar with activated ASTM class C fly ash binder, a 28-day compressive strength of 25 MPa was achievable by curing a sample underwater at 25 to 30 °C [64] without incorporating slag. Otherwise, heat curing is typically necessary to achieve reasonable mechanical properties for concrete and mortar using ASTM class F fly ash as a binder. Nonetheless, calcium in slag dissolves more readily in the activator solution to form hydration products than the calcium in class C fly ash [26]. 

**Figure 6:**
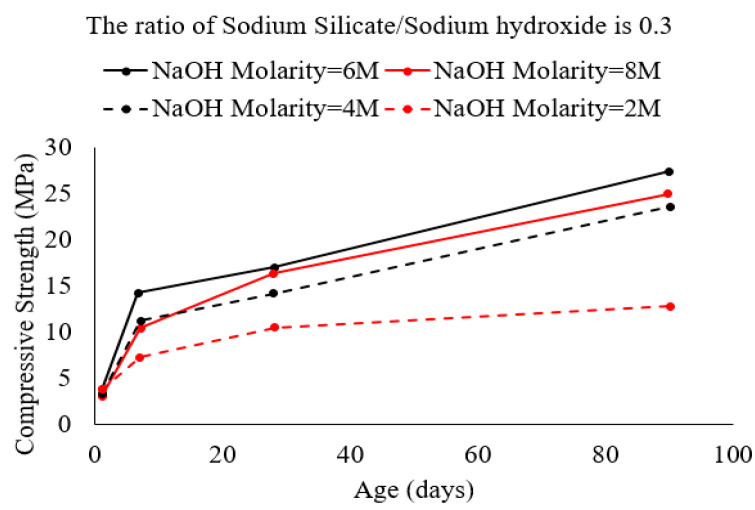
Strength development in mortar with a high-calcium fly ash binder activated using NaOH solution with molarity in the range of 2 M to 8 M [64].

The development of compressive strength in mortar with high-calcium fly ash is dependent on the molarity of the activator solution and the ratio of Na_2_SiO_3_/NaOH. When solution molarity is low (NaOH molarity = 2 M), compressive strength in high-calcium fly ash mortar is limited after the age of 28 days compared to the initial development but is highly dependent on the ratio of Na_2_SiO_3_/NaOH. However, as NaOH molarity is increased from 2 M to 8 M, long-term strength (90 days) increases significantly with increased NaOH molarity as shown in Figure 6. The data published by Ruengsillapanun et al. [64] seem to indicate that Na_2_SiO_3_/NaOH contributes significantly to early strength development at low NaOH molarity, while the increase in NaOH molarity increases strength in the long term. However, calcium has a limited direct contribution to long-term strength development in alkali-activated mortar/concrete. 

Izquierdo et al. [52] reported that 50 mm cubic mortar specimens with slag–fly ash binders, activated using potassium silicates and cured in a closed system, developed higher strength after 28 days than samples cured in an open system for the same period of time. The porosity of the geopolymerization products was higher when samples were cured in an open system compared to samples cured in a closed system. A closed curing system was represented by keeping samples in covered casting molds until the test day, while in the case of an open system, the casting molds were left uncovered. When the curing system is open, water evaporates from samples at a faster rate than the polymerization process, leading to the partial precipitation of the alkaline activator. The precipitated alkaline activator no longer contributes to the production of hydration products but is available for possible leaching. Therefore, the fundamental curing principle for alkali-activated mortar is to ensure the availability of the alkaline activator that is dissolved in water for the polymerization reaction to start and continue [2].

OPC-based 50 mm cubic mortar samples immersed in distilled water after casting developed slightly higher strength than samples that were not immersed in water (exposed), after 30 and 90 days of curing [60]. On the other hand, alkali-activated slag samples cured at 25 °C and 90% relative humidity lost some strength after 30 and 90 days of curing, but the strength increased significantly at the age of 150 days in comparison with unexposed samples.

Bernal et al. [60] reported an insignificant change in strength or porosity of mortar with an AAS binder after submersion in diluted mineral acid for 150 days. The pH of the hydrochloric, nitric, and sulfuric acids was maintained at 3.00. The limited effect of mineral acid on mortar is likely due to the low solubility of calcium salts. On the other hand, exposure to acetic acid (CH_3_COOH) with pH = 4.5 caused a degradation in strength and an increase in the volume of pores [60]. The resistance to damage due to sulfuric acid that was exhibited by mortar with an AAS binder is consistent with the data reported by Mohamed et al. [2] and was attributed to curing the sample under water that contributed to the continued formation of geopolymerization products.

**Figure 7:**
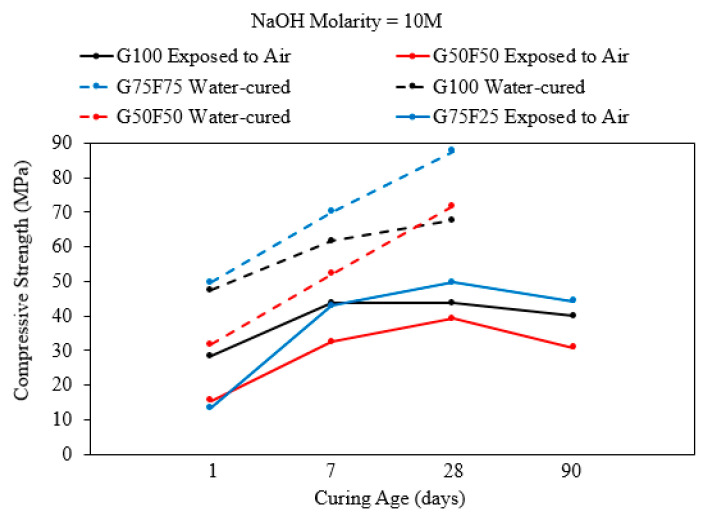
Compressive strength of mortar cured in open and closed systems for up to 90 days with an AASF binder and NaOH molarity = 10 M. Air-cured mortar decreased in strength by the age of 90 days [2].

Mortar samples with an AASF binder that were cured in air (exposed), which is an open system, experienced a degradation in compressive strength after 90 days when the binder consisted of 100% slag, 75%slag + 25% fly ash, or 50%slag + 50% fly ash [2] as shown in Figure 7. Evaporation of mixing water may have led to the shrinkage of mortar with a high GGBS content. In addition, the evaporation of water decreases the amount of activator solution that can react with the precursor to develop the binding gel in the short term. Similar samples cured in solution for 90 days did not experience any reduction in compressive strength. Instead, strength continued to increase after the 7-day compression test until the age of 90 days. Mortar cured in water must have developed a denser pore system and highly cross-linked polymerization products, especially the samples with a slag:fly ash ratio of 3:1, which developed the highest compressive strength when the NaOH activator molarity was 10 M [2].

**Figure 8:**
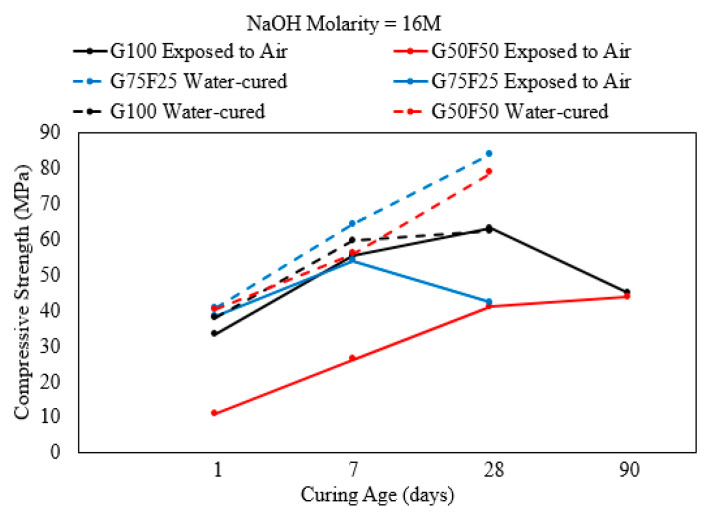
Strength development of mortar with an AASF binder cured in air or in a closed curing system with NaOH activator molarity = 16 M [2].

When the NaOH activator solution had the highest molarity (16 M), the influence of the curing system on strength evolution remained unchanged as for mortars developed with the lowest NaOH molarity (10 M) [2]. That is, samples cured in an open system (exposed to air) increased in strength from 1 to 28 days but experienced a degradation in strength by the age of 90 days as shown in Figure 8. On the other hand, the strength of samples cured in water increased consistently from the curing age of 7 to 90 days, irrespective of the binder composition. A closed curing system avails the alkaline activator solution for GGBS and fly ash to develop geopolymerization products, especially for fly ash as it needs more time to dissolve and form products. Regardless of the solution alkalinity, mortar with an AASF binder, where the slag:fly ash binder ratio was 3:1, developed the highest compressive strength after 90 days of curing in a closed system that prevented the loss of moisture through evaporation [2].

**Figure 9:**
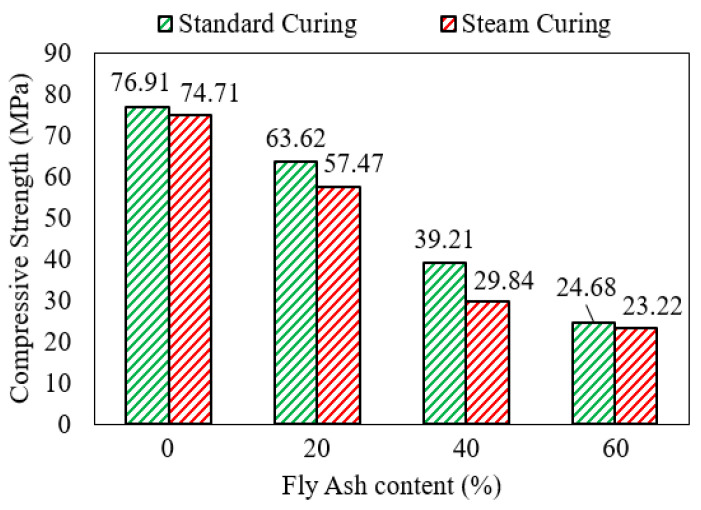
Steam curing resulted in slightly lower 28-day compressive strength than standard curing with an AASF binder for a fly ash content up to 60% [38].

Hu et al. [38] reported that mortar with an AASF binder developed relatively higher compressive strength after 28 days of water curing, in contrast with steam-cured mortar samples. The result was consistent for mortar samples with a fly ash content in the range of 0% to 60% and various alkali-activator dosages as shown in Figure 9. The slightly lower 28-day strength of steam-cured samples was attributed to the high temperature that accelerates alkali activation to the point where the distribution of reaction products and filling of the pore system become less effective [38]. Curing without steam but at a high temperature of 65 °C accelerated the development of the compressive strength of mortar with an AASF binder at an early age (1 to 7 days). By the curing age of 28 days, the strength development of similar AASF mortar samples that were cured at different initial temperatures was largely similar [65]. The early strength development under high temperatures may be due to the expedited initiation of the geopolymerization reaction and breakage of the Si-O and Al-O bonds of SiO_2_ and Al_2_O_3_ in the precursor.

## 5. Effect of the Relative Amounts of Slag and Fly Ash and the Addition of Microsilica on Mechanical Properties

The water demand of mortar/concrete decreases when fly ash is added to OPC or to slag in the case of alkali-activated binders. When AAF is used as a sole binder, mortar/concrete may require heat curing to achieve a compressive strength that is suitable for practical applications within a reasonable time after casting. On the other hand, replacing up to 30% of fly ash with slag enhances the 28-day compressive strength significantly but decreases the setting time [12,66].

In concrete/mortar with a blended slag–fly ash binder, an increase in the slag content contributed significantly to strength development at an early age when the mortar/concrete samples were cured under ambient conditions, especially when the slag content exceeded 50% [1,2]. When cured at 27 °C, the strength development of mortar was dominated by slag dissolution as C-S-H developed and precipitated, with limited or no interaction with the fly ash [67]. When the same mortar was cured at 60 °C for 4 h after curing at 27 °C for 48 h, a significant interaction between the fly ash and slag occurred, and geopolymerization products consisting of both C-S-H and A-S-H were detected in the early hours after casting. After heating at 60 °C, the gel resulting from the geopolymerization of GGBS and fly ash included C-S-H and A-S-H that coexisted to create a dense and more compact matrix. The compact matrix created by the coexistence of C-S-H and A-S-H was responsible for the rapid increase in strength as the GGBS content increased.

**Figure 10:**
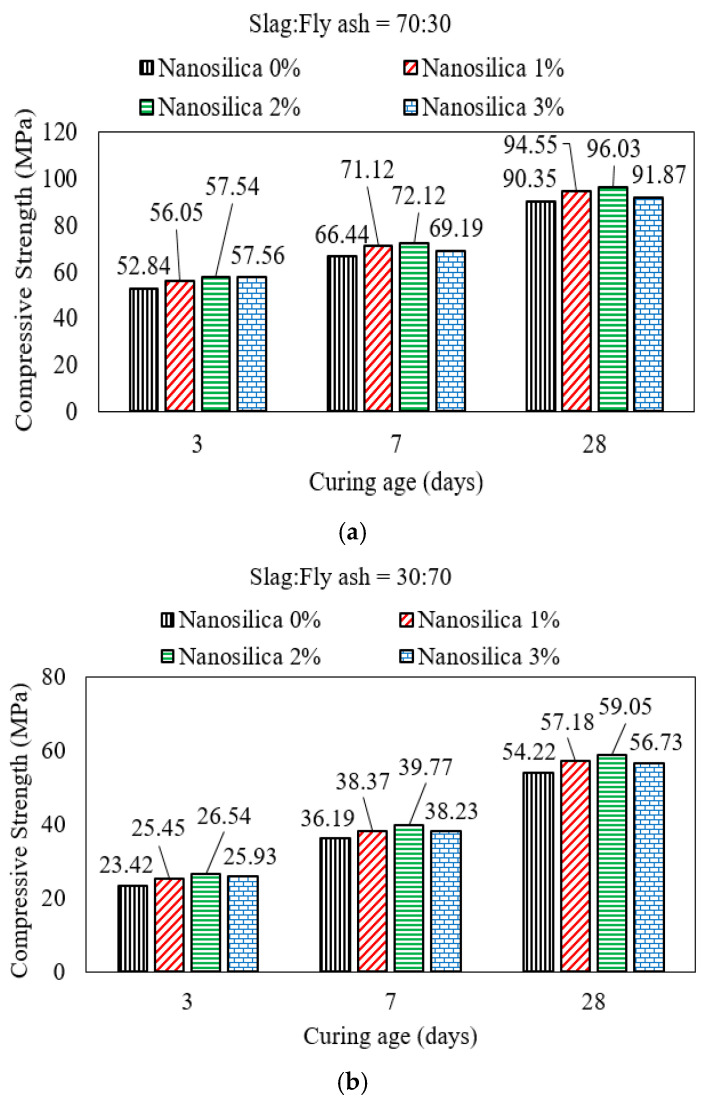
Compressive strength of mortar with AASF slag/fly ash blends. The ratio of slag:fly ash is (**a**) 70:30, (**b**) 30:70 [27].

Up to the age of 28 days, the addition of nan-silica to mortar with an AASF binder had a relatively smaller effect on strength development compared to OPC-based mortar/concrete. This is because of the overabundance of silica in fly ash, which takes a longer time to dissolve and contribute to the development of polymerization products. As a result, even with the fineness of microsilica, which supports faster dissolution and participation, an overabundance of silica outweighs the speed of dissolution of the added nano-silica. Therefore, the effect of nano-silica on strength development is even smaller when the fly ash content is high compared to slag. Gao [27] reported that adding up to 2% microsilica to mortar with an AASF binder reduced the porosity and increased the compressive strength up to the curing age of 28 days. The addition of microsilica that exceeded 2% of the total binder content decreased the compressive strength to some extent as shown in Figure 10. No notable improvement in porosity was reported with the addition of microsilica. The positive effect of adding microsilica is primarily due to the additional reactive silica that hydrates and produces a silicate–hydrate binding gel and fills the voids. A similar enhancement in compressive strength was observed in conventional concrete when an OPC binder was partially replaced with silica fume. The increase in strength after 7 and 28 days of curing accelerated with a silica fume replacement ratio of up to 15% of the total OPC binder. An increase in the silica fume content beyond this replacement ratio decreased the compressive strength of samples cured for 7 and 28 days.

The compressive strength of mortar with an AASF binder is highly influenced by the slag and fly ash proportions. However, strength development also depends on the activator concentration, activator type, and curing environment. When the activator was sodium metasilicates representing 8% of the total binder, the compressive strength of all water-cured concrete and mortar samples increased from the age of 28 days to 90 days [28].

Studies have shown that increasing the relative content of slag with respect to that of fly ash increases the Ca/Si ratio in the C(N)-A-S-H, a reaction product that characterizes alkali-activated mortar with a high slag content. The increase in the Ca/Si ratio in C(N)-A-S-H is associated with enhanced mechanical properties due the decrease in porosity and refinement of the gel microstructure [68]. The same study reported a higher compressive strength in mixes where the slag:fly ash ratio was 4:1 in contrast with lower slag-to-fly ash ratios when the activator used was anhydrous Na_2_Sio_3_ and water glass, without NaOH. It is worthy to note that samples were heat-cured after casting for 72 h, prior to preparation for further testing.

**Figure 11:**
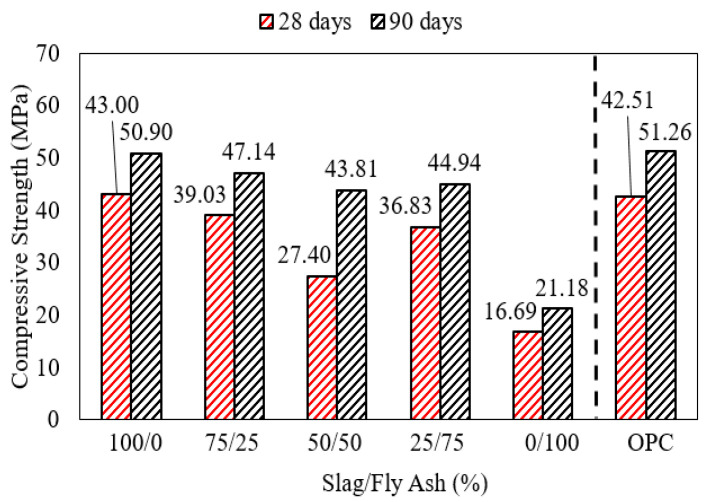
Strength development of mortar with an AASF binder. NaoH concentration = 8% for slag:fly ash ratios = 100/0, 75/25, and 50/50. NaOH concentration = 12% for the 25/75 ratio [28].

When the content of fly ash in an AASF binder increased from 0% to 50%, both the 28-day and 90-day strengths of water-cured mortar samples decreased as shown in Figure 11. Mortars prepared with 100% alkali-activated slag binders or with 75% slag + 25% slag developed comparable compressive strength after 28 and 90 days. However, mortar with a 50% slag + 50% fly ash binder exhibited a lower compressive strength at the age of 28 days in comparison with mortar with 100% slag binder [28]. This may be due to the slow reaction that is typical of ASTM class F fly ash from the early stage of curing up to 28 days. These results are similar to the findings of Mohamed [1], but the results were also dependent on the alkalinity of the activator solution. However, by the age of 90 days, mortar samples with 50% slag + 50% fly ash binders developed comparable strength to samples with a slag content higher than 50% [28]. The activator dosage is an important factor contributing to strength development under a particular combination of slag and fly ash. An optimum activator dosage can develop a binding phase consisting of calcium-substituted sodium alumino-silicate (C, N)-A-S-H characterized by high strength [69]. Therefore, combining slag with fly ash is advantageous in developing concrete/mortar at ambient conditions with particularly higher strength, which would be not possible with slag or fly ash alone. Despite the slow reactivity of fly ash, it is essential for various fresh properties, such as reducing the water demand and enhancing workability; both qualities are attributed to its particle size and morphology [70].

**Figure 12:**
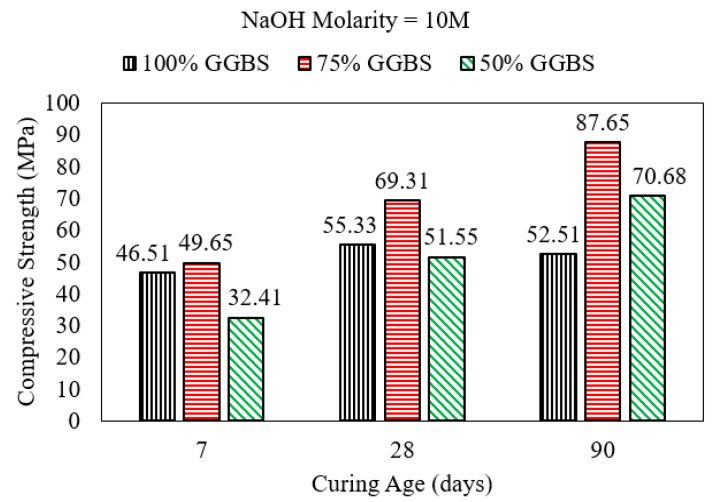
Strength development of mortar with an AASF binder immersed in sulfuric acid [6].

Mohamed [2] reported that AASF mortar samples subjected to sulfuric acid with a high slag content (≥50%) increased in compressive strength significantly by the age of 90 days in contrast with the strength after 7 days of curing. Mortar specimens in the study were submersed in sulfuric acid solution 24 h after casting, which provided a closed curing system allowing for mortar maturity in the presence of the alkaline solution [2]. As shown in Figure 12, mortar with an AASF binder where the slag:fly ash ratio was 3:1 reached a higher compressive strength than mortars with slag as a sole binder and mortar with a slag:fly ash ratio of 1:1 when the NaOH molarity was 10 M. The fact that a slag:fly ash ratio of 3:1 is optimal for enhanced mechanical properties of mortar/concrete with an AASF binder was also reported in the literature for mixes with an alkaline activator consisting of 10% Na_2_O [71]. However, Ismail et al. [28] reported a continuous degradation in the strength of samples tested after 28 days of curing as the content of fly ash in the binder increased from 0% to 50%. This difference may be due to the test parameters, such as solution alkalinity, which plays a critical role in strength development.

When the NaOH activator solution molarity was 16 M (the highest in the study), mortar with a binder comprised of equal amounts of slag and fly ash exhibited higher 90-day compressive strength than mixes containing higher GGBS contents (75% GGBS and 100% GGBS) [2]. Higher solution alkalinity in a closed curing environment enhances the dissolution and activation of fly ash and provides sufficient Na to develop polymerization products. The resulting gel system formed by the polymerization of GGBS and fly ash is highly cross-linked with a denser pore system [1].

When cured under ambient conditions, conventional concrete with a high slag replacement ratio (slag:OPC was 80:20) demonstrated a very slow rate of strength development until the age of 28 days [7]. The activation of slag is necessary before it can contribute to strength development and requires a sufficient amount of portlandite, which is produced through the hydration of OPC. The portlandite produced by the small amount of OPC binder (20% of total binder) was insufficient to activate the slag and contribute to strength development. However, for other amounts of OPC in binary GGBS/OPC mixes, it was shown that after 7 and 28 days of moist curing, the optimum OPC replacement percentage was 35% (i.e., 65% OPC and 35% GBBS), which produced a higher compressive strength than the control mix that with 100% OPC. On the other hand, replacing 80% of OPC with GGBS reduced the 7-day and 28-day compressive strength of moist-cured cubic concrete samples by 30% and 23.65%, respectively [7]. Nonetheless, the 28-day compressive strength of 80% GGBS+20% OPC blended concrete samples reached 50.45 MPa, high enough for many practical applications. This was achieved by maintaining the water-to-binder ratio (w/b) as low as 0.36 and adjusting the superplasticizer to maintain flowability and stability [7].

## 6. Use of Superplasticizers with Alkali-Activated Mortar and Concrete

Superplasticizers are water-reducing admixtures that are commonly utilized to enhance the workability and flowability of OPC-based concrete and mortar as well as those using alkali-activated binders [32]. Despite the strength and durability benefits of increasing slag content in slag–fly ash binder systems to more than 25%, significant decreases in the setting time and a decrease in mortar/concrete workability were reported [9]. Some retardation of the mortar setting time and an improvement in workability were observed in geopolymer mortar with a slag–fly ash binder when polycarboxylate-based superplasticizer was used compared to naphthalene-based superplasticizer [32].

Keulen et al. [72] indicated that the compressive strength of concrete with an AASF binder increased with the amount of the carboxylate superplasticizer, up to an optimum value of 3 to 4 kg/m^3^. The pattern was consistent for samples cured for 7, 28, and 56 days. The binder examined by the investigators consisted of 73.7 wt% class F fly ash, 25 wt% GGBS, and 1.3 wt% sodium metasilica pentahydrate powder, activated using a mixture of NaOH and Na_2_SiO_3_ solutions. The 1-day strength retardation with the increase in the superplasticizer content was consistent with the prolonged setting time typical at an early age and was observed in this study as the superplasticizer amount increased. The rapid setting of mortar/concrete was consistently reported as a shortcoming associated with an increased slag content when AASF was used as a binder [9,73].

## 7. Effect of Solution Alkalinity and Crystallinity

Manufactured slag (GGBS) contains predominantly amorphous(non-crystalline) calcium silicates that react differently with alkaline activators than other precursors where calcium is supplied in the form of crystalline silicates [74]. Typically, increasing the activator concentration increases the solution alkalinity [13]. The alkaline activation of slag in low alkalinity solutions produces C-S-H and geopolymeric gel, both of which co-exist and contribute to the formation of the binding gel and the enhancement of compressive strength development [74]. The compressive strength of slag-based mortar does not increase with an increase in solution alkalinity [74]. At higher NaOH concentrations, the effect of activator molarity on strength development also depends on the curing environment. Mohamed [1] reported that the strength of water-cured mortar with an AAS binder decreased as the concentration of the activator NaOH increased from 10 M to 16 M. A NaOH molarity of 10 M was also reported as the optimum alkali activator concentration for strength development [2,33]. This is because at higher solution alkalinity, calcium reacts fast and forms precipitates instead of developing hydrated gel [74]. Samantasinghar & Singh [75] studied NaOH concentrations in a wider range from 2 M to 16 M and reported 8 M as the optimum NaOH alkaline molarity for the highest mechanical properties of mortar with an AASF binder.

**Figure 13:**
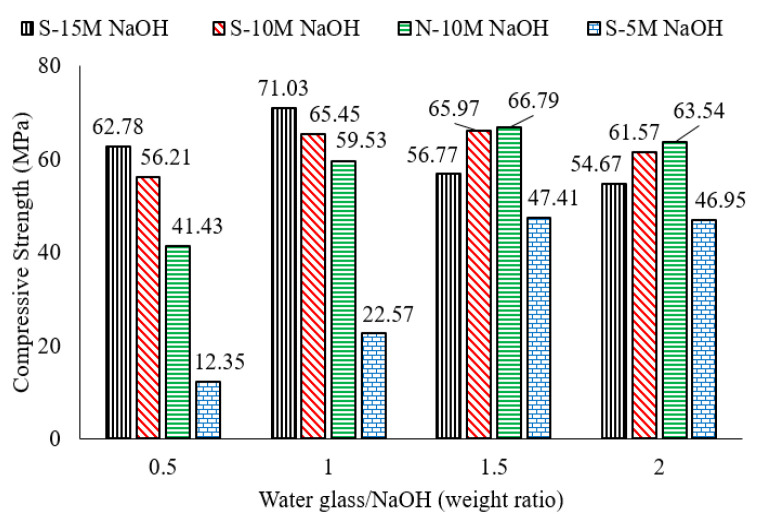
Variation of the mortar strength with the ratio of sodium silica/sodium hydroxide for AAF, using different mixing methods. Scheme S: fly ash mixed with NaOH for 10 min, followed by the addition of sodium silicate. Scheme N: fly ash, sodium silicate, and NaOH mixed simultaneously [76].

After contact with the alkaline activator, fly ash reacts slower than slag, which influences the rate of strength development. In addition, the solubility of fly ash depends on the method of mixing with alkaline activator solution(s). When fly ash was mixed with NaOH in liquid form for 10 min, followed by the addition of sodium silicate solution, high compressive strength was achieved at a lower sodium silicate/sodium hydroxide ratio (Na_2_SiO_3_/NaOH = 1.0) [76]. A higher sodium silicate/sodium hydroxide ratio (1.5) was necessary to achieve similar strength when the activator solutions NaOH and Na_2_SiO_3_ were mixed simultaneously with fly ash as shown in Figure 13 which has cost implications. In mortar with an AAF binder, increasing the concentration of the NaOH alkaline activator increases the compressive strength. This is because increasing the concentration also increases solution alkalinity, which enhances the leaching of Si and Al ions from the precursor, making them available to develop the polymerization products [76].

When fly ash-based binder was activated using a combination of NaOH and Na_2_SiO_3_ solutions, the optimum strength development of the mortar depended on the NaOH solution concentration and the ratio of Na_2_SiO_3_/NaOH. At Na_2_SiO_3_/NaOH = 1.0, strength increased with NaOH molarity, as shown in Figure 13. However, when the activator ratio Na_2_SiO_3_/NaOH = 1.5 or higher, strength did not increase with NaOH molarity. In all cases, the heat curing of AAF fly-ash-based geopolymer mortars was necessary to accelerate the compressive strength and overcome the inherently slow reactivity of the fly ash.

To evaluate its effect on the properties of geopolymer mortar/concrete, the strength of the alkaline activator is often expressed as the percentage of Na_2_O in the solution. When the content of Na_2_O in an activator consisting of sodium silicates and sodium sulfates was increased from 1.75% to 2.75%, the compressive strength of mortar with an AASF binder also increased at each curing age (1, 3, 7, and 28 days) [24]. In addition, up to 28 days, which was the maximum curing age in the study, the compressive strength increased when the curing temperature was increased from 20 °C to 40 °C [24]. Tran and Nguyen [77] reported that the highest 28-day strength of mortar with alkali-activated slag and fly ash binders was obtained when the Na_2_O concentration ranged from 4 to 6% of the total weight of the solid material in the mix.

## 8. Conclusions

This review highlighted some of the critical factors affecting the strength development of sustainable concrete/mortar with slag and fly ash binders. The main findings are listed below.

- The coexistence of geopolymerization products that are based on fly ash (N-A-S-H) and GGBS (C-A-S-H) produces a microstructure with better cross-linking and a more compact pore system. The strength development of mortar and concrete with alkali-activated GGBS-fly ash blended binders is more significant than the strength development when either of the two is used independently as an alkali-activated binder of concrete/mortar.
- The strength development of mortar with alkali-activated slag and fly ash binders exposed to acidic medium is complex and depends on multiple factors, including the relative contents of the precursors, the concentration of the alkaline activator, curing conditions, and an acidic medium. The effect of an acidic medium on the mortar/concrete strength depends on its concentration, which is related to its ability to dissociate. The effect of an acidic medium on the strength of concrete also depends on the solubility of the products of acid attack.
- A higher slag content enhances resistance to strength degradation in sulfuric acid but is less effective in acetic acid. This is attributed to the higher solubility of calcium acetate and the relativity lower solubility of calcium sulfates.
- Damage due to acid attack may lead to a substantial decrease in compressive strength. The decrease is attributed to the decalcification of silicates gel, followed by the formation and deposition of calcium-based salts, such as gypsum. The decalcified gel has poor structural properties, leading to inferior mechanical properties of the mortar/concrete. This mechanism is the primary reason OPC-based mortar/concrete performs poorly compared to mortar with alkali-activated slag or fly ash. The dealumination of the aluminate silicate hydrate, which occurs due to the ejection of aluminum when acid protons attack the polymeric Si-O-Al bonds in the aluminosilicate framework, has also been reported when mortar is subjected to attack by sulfuric acid.

## Figures and Tables

**Table 1:** Geopolymerization products of alkali-activated slag and fly ash binders.

Characteristic	Blended Slag and Fly Ash	
	Fly Ash Dominant	Slag Dominant
Main geopolymeration and hydration product.	N-A-S-H [28] dominant when fly ash ≥ 25% [35]. N-A-S-H absent from gel during first hours after casting [24].	C-A-S-H, dominant with slag ≥ 50% [35]. C-A-S-H detected within 1 day of curing [24]. C-N-A-S-H (slag 10% to 30%) [31]
Microstructure	Coarse, less compact, porous [30]. Pore diameter depends on the activator type [37].	Dense and compact [38,39]
Porosity, tortuosity, and permeable voids.	Total porosity increases with the fly ash content [38]. Volume of permeable voids increases with the fly ash content [28].	Segmented porosity decreases with an increase in slag, especially >50% [35]. Microporous zeolites absent [26]
Setting	Long setting time	Fast setting time, especially with slag > 70%
Shrinkage	Limited	High autogenous shrinkage, especially with slag > 70%

**Table 2:** Change in mass and strength due to the exposure of mortar to selected acidic solutions.

Binder	Activator Solution	Curing Condition	Acid Type	Exposure Period	Strength Change	Mass Change	Reference
100% fly ash	NaOH	Ambient for 24 h, then 95 °C for 24 h	Acetic	2 months	−45.00%	−0.45%	[37]
100% fly ash	Na_2_SiO_3_	Ambient for 24 h, then 95 °C for 24 h	Acetic	2 months	−46.00%	3.83%	[37]
100% fly ash	NaOH	Ambient for 24 h, then 95 °C for 24 h	Sulfuric	2 months	−10.40%	−1.96%	[37]
100% fly ash	Na_2_SiO_3_	Ambient for 24 h, then 95 °C 24 h	Sulfuric	2 months	−82.00%	−2.56%	[37]
100% fly ash	Na_2_SiO_3_, NaOH	Heat cured at 85 °C for 48 h, then ambient until testing	Sulfuric	2 months	−32.22%	−0.22%	[49]
100% fly ash	Na_2_SiO_3_	Ambient at 20 °C and RH 50 for 28 d	Sulfuric	28 days	−81.85%	−0.63%	[54]
100% fly ash	Na_2_SiO_3_, NaOH	Heat Cured at 80 °C for 24 h, then ambient for 28 d	Sulfuric	28 days	−18.22%	1.50%	[55]
100% fly ash	Na_2_SiO_3_, NaOH	Heat cured at 80 °C for 24 h, then ambient 28 d	Sulfuric	3 months	−33.24%	−4.25%	[55]
100% fly ash	Na_2_SiO_3_, NaOH	Ambient 23 °C & RH 100 3 d, then water cured for 28 d	Acetic	28 days	−11.06%	−3.49%	[59]
100% fly ash	Na_2_SiO_3_, NaOH	Ambient at 23 °C & RH 100 3 d, then water cured for 28 d	Acetic	3 months	−39.06%	−5.10%	[59]
90% FA 10% Slag	Na_2_SiO_3_	Ambient at 20 °C & RH 50 for 28 d	Sulfuric	28 days	−40.74%	0.69%	[54]
90% FA 10% Slag	Na_2_SiO_3_	Ambient at 20 °C & RH 50 for 28 d	Sulfuric	56 days	−51.59%	−0.11%	[54]
90% FA 10% Slag	Na_2_SiO_3_, NaOH	Ambient at 25 °C & RH 90 for 28 d	Acetic	6 months	−39.59%	−5.04%	[50]
80% FA 20% Slag	Na_2_SiO_3_, NaOH	Ambient at 25 °C & RH 90 for 28 d	Sulfuric	6 months	−49.90%	−2.54%	[50]
80% FA 20% Slag	Na_2_SiO_3_, NaOH	Ambient at 20 °C & RH 90 for 28 d	Sulfuric	56 days	−67.18%	7.46%	[9]
80% FA 20% Slag	NaOH	Ambient at RH 90 for 21 d, then water cured for 7 d	Acetic	2 months	−38.00%	−2.00%	[58]
70% FA 30% Slag	Na_2_SiO_3_	Ambient at 20 °C & RH 50 for 28 d	Sulfuric	28 days	−73.24%	−0.37%	[54]
70% FA 30% Slag	Na_2_SiO_3_	Ambient at 20 °C & RH 50 for 28 d	Sulfuric	56 days	−85.88%	−0.11%	[54]
60% FA 40% Slag	Na_2_SiO_3_, NaOH	Ambient at 20 °C & RH 90 for 28 d	Sulfuric	56 days	−62.36%	3.22%	[49]
60% FA 40% Slag	NaOH	Ambient at RH 90+ 21 d, then water cured for 7 d	Acetic	2 months	−39.00%	−2.24%	[58]
50% FA 50% Slag	Na_2_SiO_3_, NaOH	Ambient 24 h	Sulfuric	28 days	29.40%	0.85%	[1,2,5,6]
50% FA 50% Slag	Na_2_SiO_3_, NaOH	Ambient at 23 °C & RH 65 for 28 d	Sulfuric	6 months	−68.65%	−6.30%	[46]
50% FA 50% Slag	Na_2_SiO_3_	Ambient at 20 °C & RH 50 for 28 d	Sulfuric	28 days	−49.68%	−3.27%	[54]
50% FA 50% Slag	Na_2_SiO_3_	Ambient at 20 °C & RH 50 for 28 d	Sulfuric	56 days	−61.33%	−5.78%	[54]
80% FA 20% OPC	Na_2_SiO_3_, NaOH	Ambient at 25 °C & RH 90 for 28 d	Acetic	6 months	−68.67%	−8.66%	[50]
80% FA 20% OPC	Na_2_SiO_3_, NaOH	Ambient 25 °C RH 90 28 d	Sulfuric	6 months	−66.42%	−1.27%	[50]
OPC	H_2_O	Ambient for 24 h, then heat to 95 °C for 24 h	Acetic	2 months	−91.00%	−10%	[37]
OPC	H_2_O	Ambient for 24 h, then heat to 95 °C for 24 h	Sulfuric	2 months	−100.00%	>40%	[37]
OPC	H_2_O	Water cured	Acetic	3 months	−56.50%	−0.64%	[52]
OPC	H_2_O	Water cured	Sulfuric	3 months	−17.90%	−0.24%	[52]
80% OPC 20% FA	H_2_O	Ambient for 24 h, then heat to 95 °C for 24 h	Acetic	2 months	−69.00%	−5.47%	[37]
80% OPC 20% FA	H_2_O	Ambient for 24 h, then heat to 95 °C for 24 h	Sulfuric	2 months	−77.00%	19.15%	[37]
100% Slag	Na_2_SiO_3_, NaOH	Ambient for 24 h	Sulfuric	28 days	39.90%	0.88%	[1,2,5,6]
100% Slag	Na_2_SiO_3_, NaOH	Ambient at 23 °C & RH 100 for 3 d, then water cured for 28 d	Acetic	28 days	−7.29%	−3.82%	[59]

## Data Availability

No data was created in this review article.
